# Enhancer Profiling Reveals a Protective Role of RXRα Against Calcium Oxalate‐Induced Crystal Deposition and Kidney Injury

**DOI:** 10.1002/advs.202411735

**Published:** 2025-03-17

**Authors:** Yu Yang, Xudan Dou, Yongzhan Sun, Mengyao Wang, Jing Wang, Xinyi Cao, Haijie Xie, Linguo Xie, Weiping Tian, Jing Nie, Yupeng Chen, Chunyu Liu, Lirong Zhang

**Affiliations:** ^1^ Department of Urology Tianjin Institute of Urology The Second Hospital of Tianjin Medical University Tianjin 300211 China; ^2^ The Province and Ministry Co‐sponsored Collaborative Innovation Center for Medical Epigenetics Key Laboratory of Immune Microenvironment and Disease (Ministry of Education) State Key Laboratory of Experimental Hematology Department of Biochemistry and Molecular Biology School of Basic Medical Sciences Tianjin Medical University Tianjin 300070 China; ^3^ Jiangsu Key Laboratory of New Drug Research and Clinical Pharmacy Xuzhou Medical University Xuzhou Jiangsu 221004 China; ^4^ Research Center of Basic Medical Sciences Tianjin Medical University Tianjin 300070 China; ^5^ Tianjin Key Laboratory of Ionic‐Molecular Function of Cardiovascular Disease Department of Cardiology Tianjin Institute of Cardiology The Second Hospital of Tianjin Medical University Tianjin 300211 China; ^6^ Biobank of Peking University First Hospital Beijing 100034 China

**Keywords:** enhancer, epigenetics, JQ1, nephrolithiasis, RXRα

## Abstract

During the formation of kidney stones, the interaction between crystals and tubular epithelial cells (TECs) leads to tubular injury and dysfunction, which in turn promote stone formation. However, the molecular mechanisms underlying these changes in TECs remain elusive. Drug screening revealed that JQ1 inhibited the adhesion of calcium oxalate (CaOx) crystals to TECs. Its therapeutic effect is further confirmed in a glyoxylic acid‐induced CaOx crystal deposition mouse model. Utilizing epigenomic and transcriptomic profiling, dynamic enhancer landscape and gene expression program associated with nephrolithiasis are charted. Bioinformatic analysis pinpointing the RXRα as a central transcription factor (TF) modulating enhancer activity. Importantly, the animal studies revealed that RXRα deletion promoted the CaOx crystal deposition, while its activation by Bexarotene (Bex), an FDA‐approved drug, mitigated this progression. Mechanistically, under normal circumstances, RXRα inhibited nephrolithiasis‐promoting genes by recruiting the HDAC3/SMART complex to repress enhancer activity. Yet, with the progression of CaOx crystal deposition, RXRα expression decreased, leading to enhancer activation and subsequent upregulation of nephrolithiasis‐promoting genes. In summary, the work illustrates an epigenetic mechanism underlying TECs fate transition during CaOx crystal deposition and highlights the therapeutic potential of JQ1 and Bex in managing kidney stone diseases.

## Graphical abstract

Comprehensive epigenomic and transcriptomic profiling revealed a dynamic enhancer landscape and gene expression program associated with nephrolithiasis, highlighting RXRα as a central transcription factor in this regulatory network. Tubular‐specific deletion of RXRα increased intrarenal CaOx crystal deposition, while its activation with Bexarotene suppressed this process by reprogramming enhancer profiles and downregulating nephrolithiasis‐promoting genes.

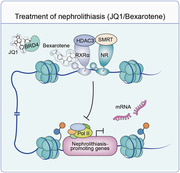

## 1 Introduction

Nephrolithiasis, commonly known as kidney stone disease, is a prevalent urological disorder associated with significant morbidity, manifesting in symptoms such as hematuria, renal colic, flank pain, urinary obstruction, and urinary tract infections^[^ ^1^, ^2^, ^3^, ^4^ ^]^ It affects ≈12% of the global population.^[^ ^5^ ^]^ Furthermore, kidney stones have a very high recurrence rate, with ≈50% of individuals experiencing a second episode within ten years of their initial occurrence.^[^ ^6^ ^]^ Additionally, the presence of kidney stones has been linked to an elevated risk of developing hypertension, diabetes, chronic kidney diseases, cardiovascular diseases, and end‐stage renal failure.^[^ ^7^, ^8^ ^]^ Existing treatments face challenges in effectively preventing the formation and recurrence of kidney stones, emphasizing the urgent need for the pursuit of innovative therapeutic approaches.

Calcium oxalate (CaOx) stones account for over 80% of urinary stones.^[^ ^9^ ^]^ The formation of CaOx stones is often associated with high urinary oxalate and/or high calcium levels. Since CaOx is only slightly soluble, an increase in urinary oxalate and/or calcium concentrations may promote the formation and deposition of CaOx crystals in the kidneys. Crystal growth and retention are critical to the pathogenesis of nephrolithiasis. Additionally, the interactions between crystals and renal tubular epithelial cells (TECs) play a pivotal role in the development of this disease.

Exposure to CaOx crystals leads to cellular injury, increases the adhesion of additional crystals to the TEC surface, and enhances crystal nucleation and retention within the kidneys.^[^ ^10^, ^11^, ^12^ ^]^ Additionally, CaOx crystals can prompt an osteogenic shift in TECs, marked by the upregulation of osteogenesis‐related genes, including *Runx2* and *Sp7*, and a rise in crucial non‐collagenous proteins such as osteopontin and osteocalcin, essential for the calcification process.^[^ ^4^ ^]^ Furthermore, crystals exposure markedly upregulates pro‐inflammatory genes in TECs, including mediators like osteopontin, matrix Gla protein, NLRP3 inflammasome, and various chemokines and cytokines, creating an inflammatory milieu that promotes stone formation.^[^ ^13^, ^14^, ^15^, ^16^ ^]^ However, the precise molecular mechanisms and key factors that govern the expression of these nephrolithiasis‐promoting genes and the fate transitions of TECs in response to CaOx crystals are still largely undefined.

Epigenetic regulation plays a central role in determining cell fate by guiding cell‐type‐specific gene expression programs.^[^ ^17^ ^]^ Various epigenetic features, including chromatin accessibility, histone modifications, DNA methylation, and non‐coding RNAs, have been linked to the gene expression programs that govern different cellular states.^[^ ^18^ ^]^ Disruptions in these epigenetic processes are implicated in the development of numerous diseases.^[^ ^19^ ^]^ In this study, we investigated the epigenetic basis of TECs fate transitions during CaOx crystal deposition. Our findings from transcriptomic and epigenomic analysis emphasize the crucial role of enhancer activation in driving nephrolithiasis‐promoting gene expression. We demonstrated the efficacy of JQ1, an enhancer‐activity‐targeting drug, in a glyoxylic acid‐induced intrarenal CaOx crystal deposition mouse model. Additionally, we delineated a transcription factor (TF) network and identified RXRα as a central TF influencing chromatin states and gene expression patterns during CaOx crystal deposition. Notably, our animal studies demonstrated that RXRα deficiency exacerbated the deposition of CaOx crystal and increased kidney injury, whereas activation of RXRα markedly reduced CaOx crystal deposition and mitigated kidney damage.

## 2 Result

### 2.1 Epigenetic Drug Screening Identifies BET Bromodomain Inhibitors in Suppressing CaOx Crystal Adhesion to TECs

**Figure 1:**
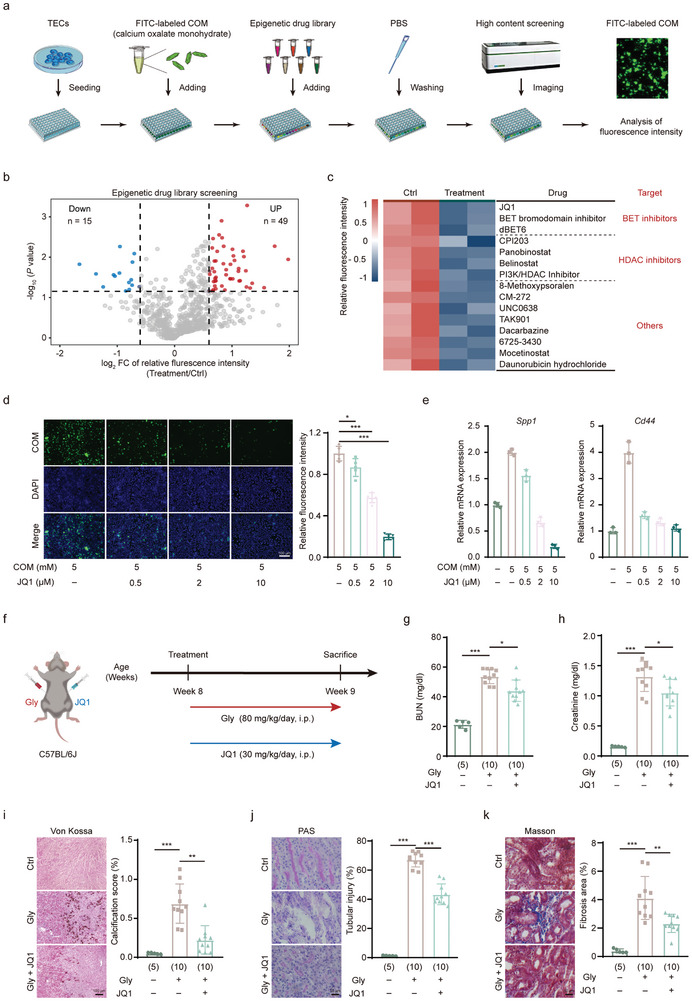
JQ1 suppresses CaOx crystal deposition both in vitro and in vivo. a) Illustration of the high‐content screening process using an epigenetic drug library of 744 epigenetic drugs in primary renal tubular epithelial cells (TECs) isolated from mouse kidneys. b) Volcano plots illustrating the effect of different drugs on cell adhesion to calcium oxalate monohydrate (COM) crystals. Red dots represent drugs that increase crystal adhesion, while blue dots represent drugs that decrease crystal adhesion (relative fluorescence log_2_ fold change > 0.6 or log_2_ fold change < −0.6, *p* < 0.05). c) Heatmap showing the 15 drugs with the greatest effect on reducing cell adhesion to COM crystals (left) and the corresponding target candidates (right). d) Analysis of FITC fluorescence intensity in primary TECs treated with different doses of JQ1 (left) and quantification of fluorescence intensity (right). e) RT‐qPCR analysis of *Spp1* (left) and *Cd44* (right) expression at the indicated doses of JQ1 treatment. f) Schematic overview of the mouse model for the formation of intrarenal CaOx crystal deposition and the treatment with JQ1. g,h) Assessment of renal function by blood urea nitrogen (BUN) and serum creatinine detected in mice with CaOx crystal deposition treated with or without JQ1. i) Von Kossa staining (left) and quantification (right) of CaOx crystal deposition mouse kidneys treated with or without JQ1, showing CaOx crystal deposition predominantly in the corticomedullary junction area. j) Injury in kidney tissue shown by PAS staining (left) and quantification (right). k) Masson's trichrome staining (left) and quantification (right) show fibrosis in renal tissues. Scale bars: 100 µm (d), 50 µm (k and j). Data are presented as mean ± SEM. ^*^ *p* < 0.05; ^**^ *p* < 0.01; ^***^ *p* < 0.001.

The adhesion of CaOx crystals to tubular epithelial cells is a crucial step in the development of nephrolithiasis. To discover drugs that inhibit this key process, we conducted a high‐throughput drug screening in isolated renal TECs incubated with FITC‐conjugated calcium oxalate monohydrate (COM) crystals. We first exposed the TECs to an epigenetic drug library consisting of 744 epigenetic drugs. This comprehensive library includes a wide range of epigenetic drugs targeting various aspects of epigenetic regulation. It comprises inhibitors and modulators of epigenetic “writers”, enzymes responsible for adding epigenetic marks; “erasers”, enzymes that remove these marks; and “readers”, proteins that recognize and bind to these epigenetic marks, thereby influencing chromatin structure and gene expression. We assessed the adhesion of COM crystals to TECs by measuring the FITC signals on the TECs (**Figure** **1**a). We identified 15 epigenetic drugs that reduced, and 49 that enhanced, the relative FITC fluorescence intensity which reflects the adherence of COM crystals to TECs surfaces (Figure 1b). Based on their mechanisms of action, these drugs can be divided into three categories: BET (Bromodomain and Extra‐Terminal motif) inhibitors, HDAC inhibitors, and others, as depicted in Figure 1c. Previous studies have reported that HDAC inhibitors prevent the formation of nephrolithiasis in animal models,^[^ ^20^, ^21^ ^]^ thereby validating our drug screening findings.

Apart from HDAC inhibitors, the drug screening results suggest that BET inhibitors also inhibit the adhesion of COM crystals to TECs. We next chose JQ1 for further analysis, as it exhibited the most pronounced inhibitory activity. JQ1 functions by targeting BET proteins, a family of epigenetic “reader” proteins. It specifically inhibits the BET bromodomains, which are responsible for recognizing acetylated lysine residues on histones. By disrupting the interaction between BET proteins and chromatin, JQ1 effectively modulates gene expression, often leading to the suppression of genes involved in various diseases, including cancer.^[^ ^22^ ^]^ We examined the effect of different concentrations of JQ1 on COM crystal adhesion to TECs. Our results showed that JQ1 reduced COM adhesion to TECs in a dose‐dependent manner (Figure 1d). Additionally, the expression of genes associated with inflammation and cell adhesion such as *Spp1* and *Cd44*,^[^ ^23^, ^24^ ^]^ exhibited a consistent decline with JQ1 administration (Figure 1e).

To evaluate the in vivo therapeutic efficacy of JQ1, we conducted further investigations into its potential in mouse models with Gly‐induced intrarenal CaOx crystal deposition (Figure 1f). JQ1 reduced the elevation of blood urea nitrogen (BUN) (Figure 1g) and serum creatinine levels (Figure 1h) induced by intraperitoneal injection (i.p.) of Gly (80 mg kg^−1^ per day), indicating a restoration of kidney function. Gly led to the deposition of CaOx within renal tubules, as evidenced by Von Kossa staining (Figure 1i). This was accompanied by pronounced damage to the renal TECs, highlighted by PAS staining (Figure 1j), and the emergence of interstitial fibrosis, as depicted by Masson's trichrome staining (Figure 1k). Remarkably, with the concurrent administration of JQ1, we observed a substantial reduction in CaOx deposition, kidney damage, and interstitial fibrosis. Additionally, JQ1 therapy mitigated the Gly‐induced upregulation of genes associated with inflammation, cell adhesion, and osteogenesis such as *Cd44* and *Spp1*, at both the mRNA and protein levels (Figure S3a,b, Supporting Information). Taken together, these results highlight a protective effect of JQ1 on TECs against CaOx crystal formation, both in vitro and in vivo.

### 2.2 JQ1 Reverses the Nephrolithiasis‐Promoting Gene Program

**Figure 2:**
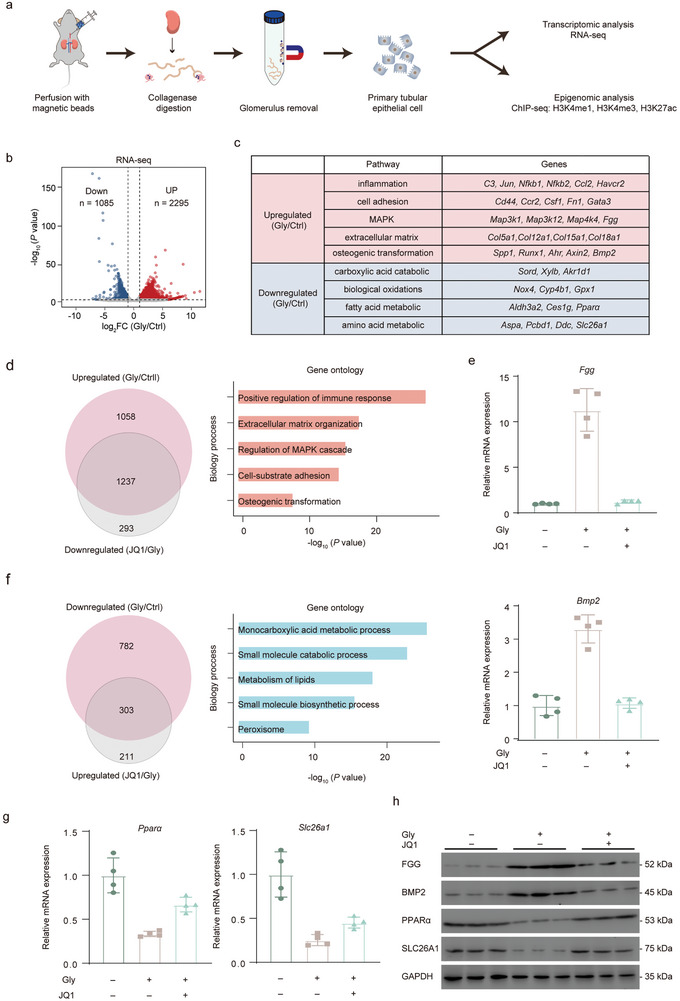
JQ1 inhibits the expression of nephrolithiasis‐promoting genes in TECs. a) Schematic overview of the isolation and multi‐omics analysis of primary TECs. b) Volcano plot of differentially expressed genes (DEG) (Ctrl versus Gly) (Ctrl: control; Gly: glyoxylic acid). Fragments per kilobase of transcript per million mapped reads (FPKM) values log_2_ fold change > 1 or log_2_ fold change < ‐1, *p* < 0.05). c) Representative genes in differential pathways associated with nephrolithiasis. d) Venn diagram illustrating the overlap of genes upregulated in Gly‐treated TECs and downregulated by JQ1 treatment (left), with Gene Ontology (GO) analysis of these genes on the right. e) RT‐qPCR validation of representative genes that upregulated in Gly‐treated TECs and downregulated by JQ1 treatment. f) Venn diagram illustrating the overlap of genes downregulated in Gly‐treated TECs and upregulated by JQ1 treatment (left), with GO analysis of these genes on the right. g) RT‐qPCR validation of representative genes that downregulated in Gly‐treated TECs and upregulated by JQ1 treatment. h) Western blot detection protein level of FGG, BMP2, PPARα, and SLC26A1 in TECs from mice treated with JQ1 or DMSO. (log_2_ fold change > 1 or log_2_ fold change < −1, *p* < 0.05). Data are presented as mean ± SEM.

Given JQ1's primary role in modulating gene expression, we next explored whether its protective effect on TECs results from restoring the gene expression program altered by CaOx deposition. To explore this, we performed transcriptomic analysis on TECs extracted from mouse kidneys under normal conditions, those treated with Gly, and those treated with both Gly and JQ1 (**Figure** **2**a).

We first compared the gene expression profiles of renal TECs from untreated mice with those from mice administered with Gly. As shown in Figure 2b, we observed an upregulation of 2295 genes and a downregulation of 1085 genes in the kidneys treated with Gly relative to the normal kidneys. Upregulated genes predominantly enriched pathways associated with osteogenic transformation, inflammation, cell adhesion, MAPK signaling, and extracellular matrix. Conversely, the downregulated genes primarily enriched pathways linked to diverse metabolic processes (Figure 2c). Integrative analysis unveiled that approximately half of the genes that were upregulated due to Gly administration were downregulated after JQ1 treatment. These downregulated genes were associated with processes including positive regulation of immune response, extracellular matrix organization, regulation of MAPK cascade, cell‐substrate adhesion, and osteogenesis (Figure 2d). We validated the transcriptomic findings with RT‐qPCR analysis of two representative genes, *Fgg* ^[^ ^25^ ^]^ and *Bmp2* ^[^ ^14^ ^]^ (Figure 2e). Furthermore, around one‐third of the genes that were initially downregulated by Gly administration were restored to their normal expression levels following JQ1 treatment (Figure 2f). We also confirmed these RNA‐seq results with RT‐qPCR of two representative metabolic genes, *Pparα* ^[^ ^26^ ^]^ and *Slc26a1* ^[^ ^27^ ^]^ (Figure 2g). Furthermore, the protein expression patterns of the aforementioned genes mirrored their mRNA expression trends (Figure 2h). Thus, these transcriptomic analyses revealed that JQ1 effectively reverses the gene expression alterations associated with nephrolithiasis in renal TECs, particularly impacting genes involved in osteogenesis, inflammatory response, cell adhesion, and various metabolic processes.

### 2.3 Active Enhancers Drive Nephrolithiasis‐Promoting Gene Expression

**Figure 3:**
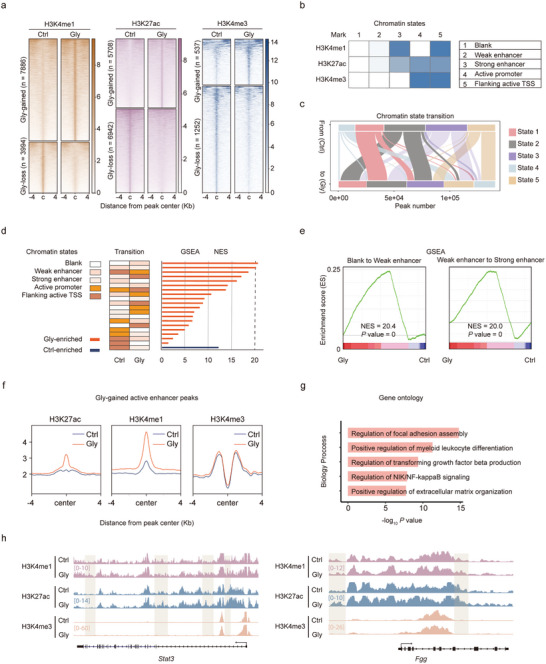
Characterization of active enhancer landscapes in Gly‐induced intrarenal CaOx crystal deposition. a) Heatmaps showing distinct enrichment of different histone modifications within the ± 4 kb region centered on the target locus. b) A color map of chromatin states calculated by ChromHMM. c) Sankey diagram illustrating the transition of chromatin states from the Ctrl to the Gly treatment. Peak length = 200 bp. d) Heatmaps showing the chromatin state transition from the Ctrl group to the Gly group (left). Gene set enrichment analysis (GSEA) of genes associated with chromatin transition (right). e) GSEA analysis of two state transitions most strongly correlated with gene expression: from Blank to Weak enhancer (left) and from Weak enhancer to Strong enhancer (right). f) Average ChIP‐seq signals are shown for different histone marks around active enhancers. g) Gene Ontology (GO) analysis of active enhancers. h) ChIP‐seq track profiles of H3K27ac, H3K4me3, and H3K4me1 on the genomic regions proximal to *Stat3* (left) and *Fgg* (right).

JQ1 modulates gene expression primarily by disrupting the interaction of bromodomain proteins with acetylated histones, particularly targeting the binding at the enhancer marker H3K27ac modification, which results in the suppression of gene transcription. To decipher the epigenetic changes and mechanisms of JQ1 in the development of CaOx crystal deposition, we carried out enhancer profiling with ChIP‐seq analysis focusing on various histone modifications, including H3K4me3, H3K4me1, and H3K27ac. As depicted in **Figure** **3**a, we observed a great number of genomic regions with marked differences in the enrichment of these histone modifications (fold change > 1.5, *p* < 0.05) in TECs treated with Gly compared to normal TECs. This observation implies a substantial alteration in enhancer landscapes within TECs during CaOx crystal deposition progression.

Diverse histone modifications cooperate to govern the chromatin state, which ultimately determines gene expression. Utilizing the ChromHMM algorithm^[^ ^28^ ^]^ to integrate the ChIP‐seq data of the above histone modifications, we annotated five distinct chromatin states across the entire genome. Here, H3K4me1 served as a marker for enhancers, H3K4me3 served as a marker for promoters, and H3K27ac served as a marker for active enhancers and active promoters. These states exhibited characteristics associated with blank regions, weak enhancers, strong enhancers, active promoters, active TSS, and flanking active TSS (Figure 3b). Notably, we observed a dynamic shift among these chromatin states in TECs during CaOx crystal deposition, particularly the shift from an inactive state to a weak enhancer state (from state 1 to state 2) and from a weak enhancer state to a strong enhancer state (from state 2 to state 3) (Figure 3c). These findings demonstrate profound dynamic changes in enhancer profiles in CaOx crystal deposition.

To integrate with gene expression, we performed Gene Set Enrichment Analysis (GSEA) analysis. This analysis unveiled a strong association between the changes in enhancer states and the alterations in gene expression (Figure 3d). The shifts from a blank state to a weak enhancer state and from a weak enhancer state to a strong enhancer state are significantly correlated with gene expression (Figure 3e). We designated the genomic regions displaying elevated enhancer markers (H3K27ac or H3K4me1) in TECs treated with Gly compared to control TECs as “Gly‐gained active enhancers” (Figure 3f). Genes associated with these Gly‐gained active enhancers were enriched in processes including regulation of focal adhesion assembly, regulation of NIK/NF‐kappaB signaling, regulation of transforming growth factor beta production, and positive regulation of extracellular matrix organization (Figure 3g), which are involved in crystal deposition, inflammation, and fibrosis.^[^ ^29^, ^30^, ^31^ ^]^ Figure 3h displays the epigenetic characteristics of several representative genes associated with Gly‐gained active enhancers. These genes include *Stat3* and *Fgg*, which are involved in extracellular matrix organization and crystal formation.^[^ ^25^, ^32^ ^]^ Taken together, these findings suggest that the active enhancers emerging after Gly treatment play a key role in activating nephrolithiasis‐associated genes.

### 2.4 Motif Analysis Reveals the Enrichment of RXRα at Active Enhancers

**Figure 4:**
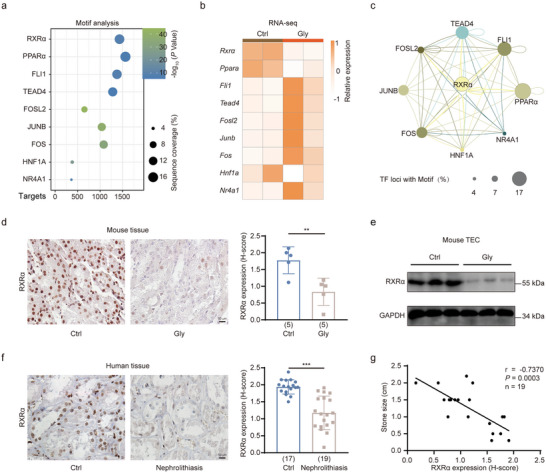
RXRα enrichment at active enhancers of nephrolithiasis‐promoting genes. a) Bubble plot illustrating the enrichment analysis of TF motifs on Gly‐gained active enhancers. b) Heatmap showing the relative expression of the enriched TFs. c) Analysis of the regulatory network of the TFs in (b). d) Immunohistochemistry staining of RXRα in kidney tissues from Ctrl and Gly‐treated mice. e) Western blotting of RXRα in renal TECs of Ctrl and Gly‐treated mice. f) Immunohistochemistry staining of RXRα in kidney tissues from Ctrl and nephrolithiasis patients. g) Correlation between RXRα abundance and stone size in patients with nephrolithiasis. Pearson's correlation coefficients are shown in the graph. Scale bars: 50 µm (d and f). *p* values were determined by linear regression analysis. Data are presented as mean ± SEM. ^**^ *p* < 0.01. ^***^ *p* < 0.001.

Transcription factors (TFs) play a dominant role in establishing and maintaining chromatin states, which in turn regulate gene expression. To identify key TFs involved in modulating chromatin states of TECs during CaOx crystal deposition, we searched for enriched TF motifs within the Gly‐gained active enhancers. This analysis unveiled nine TF motifs, with RXRα emerging as the highest‐ranked TF (**Figure** **4**a). Subsequently, we examined the gene expression profiles of these TFs and noted that nine of them displayed a change exceeding 1.5‐fold following Gly treatment (Figure 4b). Notably, PPARα was identified among these TFs, known for its role in modulating oxidative stress and calcium ion dynamics.^[^ ^33^ ^]^ Additionally, TEAD4 was also highlighted, and recognized for its involvement in the induction of osteopontin.^[^ ^34^ ^]^

TFs often collaborate to establish regulatory networks, leading to synergistic and cooperative control over gene expression programs.^[^ ^35^, ^36^ ^]^ Using the Homer algorithm,^[^ ^37^ ^]^ we mapped the TF regulatory network associated with Gly‐gained enhancers. As shown in Figure 4c, this analysis revealed that RXRα emerged as the most prominent node of this TF regulatory network. Therefore, we focused on RXRα for subsequent analysis.

### 2.5 Loss of RXRα Exacerbates Kidney Injury and CaOx Crystal Deposition

The above transcriptomic analysis indicated a decline in *Rxrα* mRNA expression, we next investigated whether its protein levels were also downregulated. As shown in Figure 4d,e, both IHC staining and WB analysis revealed a pronounced decrease in RXRα abundance in mice treated with Gly compared to normal mice. Likewise, we observed a substantial reduction in RXRα protein levels within the renal tubular cells of patients with nephrolithiasis as compared to controls (Figure 4f). Moreover, a correlation analysis revealed an inverse association between RXRα expression levels and the size of kidney stones (Figure 4g).

**Figure 5:**
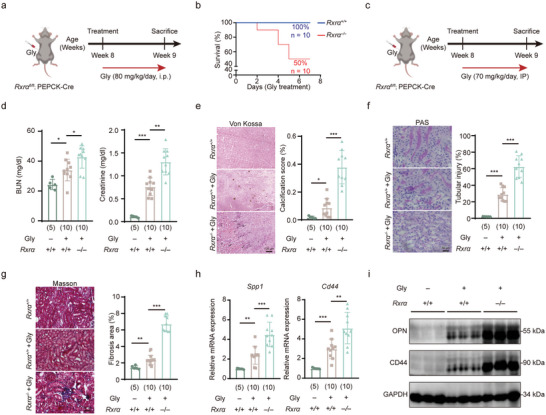
Renal tubule‐specific deletion of *Rxrα* accelerates TECs injury and crystal deposition. a) Schematic overview of the mouse model of CaOx crystal deposition in renal tubule‐specific *Rxrα* knockout mice (Gly dose: 80 mg kg^−1^ per day). b) Kaplan–Meier survival curves for *Rxrα^−/−^* mice following Gly administration. n  =  10 in each group. c) Schematic overview of the mouse model of CaOx crystal deposition in renal tubule‐specific *Rxrα* knockout mice (Gly dose: 70 mg kg^−1^ per day). d) Assessment of renal function by measurement of BUN (left) and serum creatinine (right) levels in *Rxrα^+/+^* or *Rxrα^−/−^* mice treated with Gly or saline. e) Von Kossa staining (left) and quantification (right) of kidney sections from *Rxrα^+/+^* or *Rxrα^−/−^* mice treated with Gly or saline. f) PAS staining (left) and quantification (right) of kidney tissues from *Rxrα^+/+^* or *Rxrα^−/−^* mice treated with Gly or saline. g) Masson's trichrome staining (left) and quantification (right) of kidney tissues from *Rxrα^+/+^* or *Rxrα^−/−^* mice treated with Gly or saline. h) RT‐qPCR analysis of Spp1 (left) and Cd44 (right) expression in the indicated groups. i) Western blot of OPN and CD44 in TECs from *Rxrα^+/+^* or *Rxrα^−/−^* mice treated with Gly or saline. Scale bars: 100 µm (f); 50 µm (g and h). Data are presented as mean ± SEM. ^*^ *p* < 0.05; ^**^ *p* < 0.01; ^***^ *p* < 0.001.

To explore the role of RXRα in the progression of CaOx crystal deposition, we first assessed the effect of RXRα depletion in the intrarenal CaOx crystal deposition mouse model. We crossbred *Rxrα^fl/fl^* mice with PEPCK‐Cre mice to generate tubule‐specific *Rxrα* knockout mice (*Rxrα* ^–/–^). We observed that *Rxrα^–/–^* mice displayed a heightened sensitivity to Gly administration in comparison to WT mice. Notably, an 80 mg kg^−1^ per day Gly injection (**Figure** **5**a) resulted in significant mortality exclusively in *Rxrα^–/–^* mice, but not in WT mice (Figure 5b). To mitigate survival bias for subsequent analysis, we reduced the Gly injection dosage to 70 mg kg^−1^ per day. This adjustment aimed to induce a milder form of injury to enhance the survival rate among *Rxrα^–/–^* mice (Figure 5c).

As depicted in Figure 5d,e, we noted that the *Rxrα^–/–^* mice exhibited an exacerbated increase in Gly‐induced BUN and serum creatinine levels (Figure 5d), indicating a pronounced decline in renal function. Furthermore, Von Kossa staining revealed an elevated deposition of Gly‐induced CaOx crystals in the kidneys of *Rxrα^–/–^* mice in comparison to WT mice (Figure 5e). Additionally, PAS staining (Figure 5f) and Masson staining (Figure 5g) showed that the renal injury and fibrosis induced by Gly treatment were more severe in *Rxrα^–/–^* mice when compared to WT mice. Moreover, the loss of *Rxrα* also heightened the Gly‐induced upregulation of *Cd44* and *Spp1*, genes associated with inflammation, cell adhesion, and osteogenesis (Figure 5h). Consistently, *Rxrα* depletion further increased the protein levels of OPN (encoded by *Spp1*) and CD44 (encoded by *Cd44*) (Figure 5i). Taken together, these findings suggest that tubule‐specific depletion of RXRα accelerates the progression of kidney injury and CaOx crystal deposition.

### 2.6 Bexarotene Alleviates Kidney Injury and CaOx Crystal Deposition by Activating RXRα in TECs

**Figure 6:**
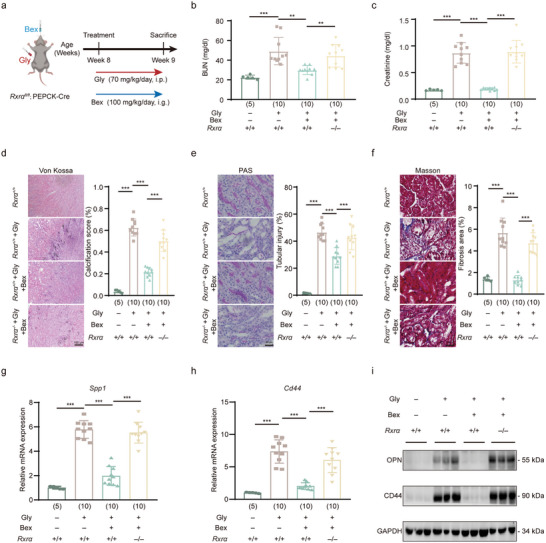
Bexarotene suppresses CaOx crystal deposition by activating RXRα. a) Schematic overview of the mouse model of CaOx crystal deposition and Bex treatment in *Rxrα^+/+^* or *Rxrα^−/−^* mice. i.g.:intragastric gavage. b,c) Assessment of renal function by measuring BUN and serum creatinine levels in Rxrα+/+ or Rxrα‐/‐ mice treated with Bex or corn oil. d) Von Kossa staining (left) and quantification (right) of kidney sections from *Rxrα^+/+^* or *Rxrα^−/−^* mice treated with Bex or corn oil. e) PAS staining (left) and quantification (right) of kidney tissues from *Rxrα^+/+^* or *Rxrα^−/−^* mice treated with Bex or corn oil. f) Masson's trichrome staining (left) and quantification (right) of kidney tissues from *Rxrα^+/+^* or *Rxrα^−/−^* mice treated with Bex or corn oil. g,h) RT‐qPCR analysis of *Spp1* (g) and *Cd44* (h) expression in the indicated groups. i) Western blotting of OPN and CD44 in TECs from *Rxrα^+/+^* or *Rxrα^−/−^* mice treated with Bex or corn oil. Scale bars: 100 µm (d); 50 µm (e and f). Data are presented as mean ± SEM. ^*^ *p* < 0.05; ^**^ *p* < 0.01; ^***^ *p* < 0.001.

To further investigate the role of RXRα in CaOx crystal deposition, we next assessed whether activating RXRα could alleviate CaOx crystal deposition. For this purpose, we employed an FDA‐approved RXRα agonist, Bexarotene (Bex), in the Gly‐induced mouse model of intrarenal CaOx crystal deposition (**Figure** **6**a). As depicted in Figure 6b,c, the administration of Bex ameliorated the Gly‐induced increase in BUN level (Figure 6b) and serum creatinine level (Figure 6c), indicating an improvement in renal function. Importantly, the absence of RXRα eliminated the protective effect of Bex on renal function (Figure 6b,c).

Moreover, Bex treatment reduced the deposition of Gly‐induced CaOx crystals in the kidneys, as demonstrated by Von Kossa staining (Figure 6d). Additionally, PAS (Figure 6e) and Masson (Figure 6f) staining revealed that Bex treatment ameliorated Gly‐induced renal injury and fibrosis. Consistently, these beneficial effects of Bex on the intrarenal CaOx crystal deposition mouse model were also abolished in *Rxrα^–/–^* mice (Figure 6d–f). Furthermore, Bex reversed the Gly‐induced upregulation of *Cd44* and *Spp1* genes, both at the mRNA and protein levels, which was not observed in the absence of RXRα (Figure 6g–i). In summary, these findings collectively suggest that Bex inhibits CaOx crystal deposition and offers renal protection via RXRα‐dependent pathways.

### 2.7 Bex‐Mediated RXRα Activation Inhibits Nephrolithiasis‐Promoting Genes

**Figure 7:**
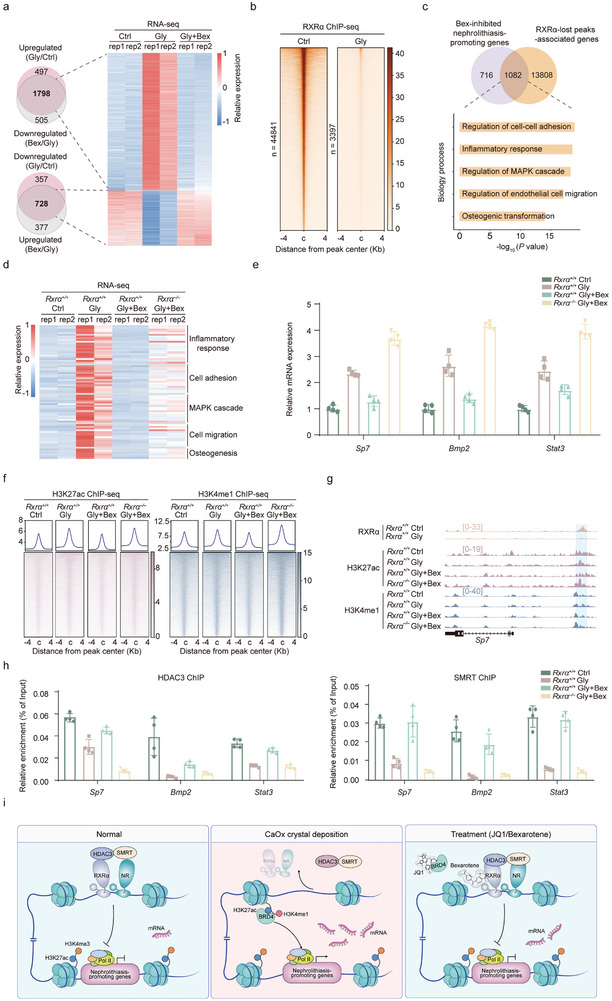
RXRα suppresses nephrolithiasis‐promoting genes by recruiting the SMRT/HDAC3 co‐repressor complex. a) Venn diagram showing the overlap of DEGs in the indicated groups and heatmap depicting gene expression in TECs from the indicated groups. b) Heatmap showing ChIP‐seq signals for RXRα binding in the indicated groups. c) Overlap analysis of the identified nephrolithiasis‐promoting genes from (a) the genes associated with RXRα‐bound genomic regions in (b) (top) and GO analysis of these 1082 genes (bottom). d) Heatmap illustrating the effect of *Rxrα* knockout on the expression of Bex‐suppressed nephrolithiasis‐promoting genes. e) RT‐qPCR analysis of *Sp7* (left), *Bmp2* (middle), and *Stat3* (right) expression in the indicated groups. f) Heatmaps illustrate the enrichment of H3K27ac and H3K4me1 modifications on genomic regions associated with genes identified in (c). g) ChIP‐seq tracks of RXRα, H3K4me1, and H3K27ac on the *Sp7* gene. h) ChIP‐qPCR of SMRT (upper) and SMRT (lower) at *Sp7*, *Bmp2*, and *Stat3* gene loci in TECs isolated from the indicated groups. i) Working model illustrating how RXRα suppresses enhancer activity in nephrolithiasis‐promoting genes, subsequently preventing the pathological transition of TECs. Data are presented as mean ± SEM.

The data presented above indicate that Bex treatment effectively delays kidney injury and CaOx crystal deposition progression. To understand how Bex regulates nephrolithiasis‐associated gene program, we conducted a transcriptomic analysis of CaOx crystal deposition mice with Bex treatment. This analysis showed that ≈75% of the genes upregulated by Gly treatment (1798 out of 2295) were downregulated following Bex treatment (**Figure** **7**a). Additionally, ≈66% of the genes initially suppressed by Gly (728 out of 1058) returned to their normal levels after Bex administration (Figure 7a). As previously demonstrated in Figure 2c, genes upregulated following Gly treatment enriched nephrolithiasis‐promoting pathways, including immune response, extracellular matrix organization, MAPK cascade regulation, cell‐substrate adhesion, and osteogenesis. Furthermore, experiments with *Rxrα* knockout mice suggest that the effectiveness of Bex in delaying nephrolithiasis progression depended on RXRα. Therefore, our subsequent analysis concentrates on exploring how the activation of RXRα by Bex inhibits the expression of these nephrolithiasis‐promoting genes.

TFs bind to the regulatory regions and recruit transcriptional activation or repression complexes to control target gene expression. Therefore, we first carried out a ChIP‐seq analysis to assess the genome‐wide binding of RXRα in TECs of both normal and Gly‐treated mice. In normal TECs, RXRα bound to 44 841 genomic regions (Figure 7b), but this binding dramatically decreased in Gly‐treated TECs, with only 3397 regions showing RXRα binding (Figure 7b). This reduction corresponds with the substantial decrease in RXRα expression in Gly‐treated TECs compared to normal (Figure 4d,e). The downregulated peaks in Gly‐treated TECs corresponded to 14 892 genes. Integrating these ChIP‐seq data with RNA‐seq data, we identified 1082 nephrolithiasis‐promoting genes as Bex‐inhibited/RXRα‐bound genes (Figure 7c). GO analysis of these genes showed marked enrichment in pathways related to nephrolithiasis, including immune response, cell adhesion, cell migration, MAPK cascade regulation, and osteogenesis (Figure 7c). These findings indicate that RXRα activation following Bex treatment suppresses nephrolithiasis‐promoting genes.

### 2.8 RXRα Recruits SMRT/HDAC3 Co‐Repressor Complex to Suppress Enhancer Activity of Nephrolithiasis‐Promoting Genes

Our prior epigenomic analysis revealed that abnormal enhancer activation activates the expression of genes promoting CaOx crystal deposition. Transcriptomic analysis of *Rxrα* knockout mice confirmed that the reversal effect of Bex on the expression of these genes is dependent on RXRα (Figure 7d). RT‐qPCR was performed to validate the expression of three representative genes—*Sp7*, *Bmp2*, and *Stat3*—which were upregulated following Gly treatment and downregulated upon Bex treatment. Additionally, RXRα knockout markedly attenuated Bex‐mediated inhibition of their expression (Figure 7e).

Next, we proceeded to examine the changes in enhancer activation markers, specifically H3K27ac and H3K4me1, on those Bex‐inhibited/RXRα‐bound nephrolithiasis‐promoting genes after Bex treatment. As shown in Figure 7f, Bex effectively reversed the activation of these enhancers. Importantly, this reversal was not observed in the absence of RXRα, indicating that Bex reshapes enhancer landscapes through RXRα activation. The representative track profiles of RXRα binding and histone modifications on *Sp7* gene were depicted in Figure 7h.

RXRα inhibits gene transcription through interactions with transcriptional corepressors, specifically the nuclear receptor corepressor (NCoR) and the silencing mediator for retinoic acid and thyroid hormone receptor (SMRT).^[^ ^38^, ^39^ ^]^ RXRα binds to SMRT, facilitating the recruitment of HDAC3 and consequently forming a corepressor complex.^[^ ^40^ ^]^ This RXRα/SMART/HDAC3 transcriptional repression complex plays a crucial role in the deacetylation of histone H3K27, thereby repressing target gene expression. To investigate whether RXRα recruits SMRT and HDAC3, we conducted ChIP‐qPCR experiments to assess the binding of SMRT, and HDAC3 on the representative Bex‐inhibited/RXRα‐bound nephrolithiasis‐promoting genes in TECs. As shown in Figure 7h, we detected the binding of HDAC3 and SMRT to the enhancers of *Sp7*,^[^ ^41^ ^]^ *Bmp2*,^[^ ^14^ ^]^ and *Stat3* ^[^ ^42^ ^]^ genes. However, this recruitment was greatly reduced in TECs treated with Gly. Importantly, treatment with Bex effectively countered the Gly‐induced reduction in recruitment. Notably, this rescue effect of Bex was not observed in TECs from *Rxrα* knockout mice (Figure 7h). Taken together, the results indicate that RXRα recruits SMRT/HDAC3 co‐repressor complex to suppress enhancer activity of genes promoting kidney injury and CaOx crystal deposition.

## 3 Discussion

Kidney stones are formed when numerous tiny crystals, combined with organic material, stick together. Crystal formation in the kidneys is considered to be normal and harmless as long as the crystals are excreted in urine. The difference in stone‐forming individuals is that these crystals remain in the kidneys instead of being expelled. Therefore, crystal deposition is the key step in stone formation. The interaction between crystals and TECs is critical for crystal deposition.^[^ ^4^ ^]^ TECs experience a profound shift in their cellular state following exposure to urinary crystals, leading to a reduction in multiple metabolic pathways while simultaneously activating pathways related to inflammation, cell adhesion, and osteogenesis.^[^ ^14^, ^43^, ^44^ ^]^ These alterations in TECs facilitate the adhesion of more crystals to the TEC surface, enhancing crystal retention in the kidneys and ultimately contributing to kidney stones, nephrocalcinosis, and kidney damage.^[^ ^45^ ^]^ Consistent with previous studies,^[^ ^46^, ^47^ ^]^ our transcriptomic analysis of isolated primary TECs revealed a profound downregulation of genes associated with normal tubular metabolic functions, indicating tubular dysfunction after crystal‐induced injury. Meanwhile, we also observed a marked upregulation of genes related to nephrolithiasis‐promoting pathways, including inflammation response, adhesion, and osteogenic transformation. These findings suggest that changes in the expression of nephrolithiasis‐associated genes within TECs promote the development of kidney injury and CaOx crystal deposition.

Growing evidence points to the pivotal role of epigenetic regulation in orchestrating cell identity gene expression programs and determining cell fates.^[^ ^48^ ^]^ Previous research has highlighted the involvement of two microRNAs, miRNA‐93‐5p and miRNA‐34a, in the pathogenesis of CaOx nephrolithiasis. Their dysregulation contributes to inflammation and enhances cellular adhesion, pivotal in crystal formation.^[^ ^49^, ^50^ ^]^ In another study, vinegar displayed efficacy in preventing renal CaOx crystal formation, achieved by modulating urinary citrate and calcium excretion. This protective effect is attributed to the enhanced acetylation of histone H3K9 and H3K27 in renal tubular cells, which promotes the expression of microRNAs‐130a‐3p, ‐148b‐3p, and ‐374b‐5p.^[^ ^51^ ^]^ Importantly, it has been reported that LncRNA H19 is significantly upregulated in Randall's plaques, activating the Wnt/β‐catenin signaling pathway to enhance osteogenic differentiation of human renal interstitial fibroblasts.^[^ ^52^ ^]^ Additionally, LncRNA H19 can interact with miR‐216b, acting via the HMGB1/TLR4/NF‐κB signaling pathway to promote oxidative stress and renal tubular epithelial cell injury induced by CaOx crystal.^[^ ^53^ ^]^ In the present work, to explore potential interventions for nephrolithiasis, we initiated a drug screening to identify epigenetic drugs capable of alleviating the adhesive state of TECs following exposure to CaOx crystals. Our screening identified BET family inhibitors, including JQ1, which effectively reduces the adhesion of CaOx crystals to TECs following crystal exposure. JQ1 is recognized for its ability to suppress the expression of disease‐related genes by disrupting the interaction between enhancers and bromodomain proteins. These findings suggest that modulating enhancer activities and chromatin states is an effective approach to reducing CaOx crystal deposition. To investigate the underlying epigenetic mechanisms, we conducted comprehensive genome‐wide profiling of several key enhancer‐related histone modifications in primary TECs isolated from the intrarenal CaOx crystal deposition mouse model. Our analysis revealed substantial enhancer reprogramming in TECs, showing that genes associated with newly acquired enhancers were highly enriched for those linked to CaOx crystal deposition, including inflammation, cell adhesion, and osteogenesis. These results provide an epigenetic basis for the increased expression of genes that contribute to crystal deposition in TECs following exposure to CaOx.

Transcription factors play a key role in the establishment and maintenance of epigenetic modifications.^[^ ^54^ ^]^ By integrating transcriptomic and epigenomic analysis, we identified RXRα as a key regulator involved in enhancer establishment. Our study shows that activation of RXRα can effectively reduce CaOx crystal deposition by downregulating genes that facilitate nephrolithiasis. Renal tubular epithelial cell‐specific knockout of RXRα exacerbated CaOx crystal deposition and abolished the therapeutic effect of Bex, suggesting RXRα activation in renal TECs is a key mechanism underlying Bex's therapeutic effect. Additionally, we found that RXRα recruited the SMART/HDAC3 co‐repressor complex to inhibit the transcription of these nephrolithiasis‐promoting genes. CaOx treatment reduced RXRα expression and derepressed SMART/HDAC3 suppressors, leading to enhancer activation and increased expression of those genes that promote nephrolithiasis. In contrast, Bex‐mediated activation of RXRα suppressed these genes by reprogramming their enhancer landscapes. These findings establish RXRα as a crucial factor in suppressing enhancer activity of genes involved in nephrolithiasis progression and in preventing the pathological cell state transition of TECs in CaOx crystal deposition (Figure 7i).

It is well established that RXR forms heterodimers with various nuclear receptors, orchestrating a complex regulatory network that influences gene expression across a broad spectrum of physiological processes.^[^ ^55^ ^]^ This includes the regulation of genes associated with calcium/phosphate metabolism and bone formation, particularly when RXR heterodimerizes with the Vitamin D Receptor (VDR).^[^ ^56^ ^]^ In our study, we observed that RXRα, in the presence of its agonists, can suppress genes associated with an osteogenic shift in renal TECs. This mirrors the gene regulation patterns seen in the RXR/VDR complex, which plays a critical role in renal calcium handling—a key factor in kidney stone pathology.^[^ ^57^ ^]^ The interaction between RXRα and VDR and their impact on nephrolithiasis‐promoting genes warrant further investigation.

The dynamic changes in the chromatin state modulate the expression of numerous genes and the activity of various signaling pathways. Therefore, targeting global chromatin states in disease treatment could potentially offer more effectiveness on disease‐related gene expression programs than focusing on individual disease‐associated genes or isolated signaling pathways. Our transcriptomic findings reveal that during CaOx crystal deposition, TECs undergo extensive transcriptional changes. These alterations impact a wide array of cellular pathways, including injury, inflammatory response, adhesion, cell cycle changes, and osteogenic transformation. JQ1, by modulating enhancer activity, reprograms the expression of multiple nephrolithiasis‐promoting gene programs and pathways, potentially achieving better therapeutic outcomes than single‐target treatments.

JQ1 is the first BET inhibitor to be widely studied and applied in preclinical research. Currently, it is being tested in clinical trials across multiple areas, with a primary focus on cancer treatment (especially hematologic cancers), immune system disorders, neurodegenerative diseases, and viral infections.^[^ ^58^, ^59^ ^]^ Since the discovery of JQ1, many other BET inhibitors have also been developed, such as CPI‐0610 and ABBV‐744, which are currently being tested in clinical trials for cancers and other diseases, including frontotemporal dementia, neuroinflammation, and inflammation‐induced cardiac dysfunction.^[^ ^60^, ^61^, ^62^, ^63^ ^]^ The advancements in the pharmacology of BET inhibitors and their current evaluation in clinical trials may expand the applications of BET inhibitors beyond cancer therapy. Our work underscores the potential of applying BET inhibitors to manage nephrolithiasis, expanding their applications in kidney diseases.

RXRα, through interactions with various transcription factors, forms a transcription factor network. Therefore, activating RXRα with Bex can reshape enhancers and regulate the expression of many nephrolithiasis‐associated genes. Bex is an FDA‐approved oral medication that has been used for over 20 years to treat skin T‐cell lymphoma, and no serious side effects have been observed in previous animal and human studies.^[^ ^64^ ^]^ In this study, we used a glyoxylic acid‐induced nephrolithiasis mouse model, which simulates pathological conditions associated with elevated oxalate levels, including primary hyperoxaluria, acquired enteric hyperoxaluria, high oxalate diet, excessive vitamin C intake, and ethylene glycol exposure. Moreover, this model primarily reflects early pathological changes related to CaOx crystal deposition, including tubular injury and inflammation. These processes may represent critical initial events that occur before patients progress to clinically apparent stone formation. The demonstrated potential of these enhancer‐targeting therapeutic strategies warrants further clinical research to verify their effectiveness in patients with nephrolithiasis. Moreover, our data, which correlate human RXRα expression levels with crystal occurrence and size, underscore the clinical relevance of our findings and highlight the potential for RXRα modulation as a promising therapeutic avenue in managing nephrolithiasis.

## 4 Experimental Section

### Human Subjects

Kidney tissues for this study were sourced from patients with kidney cancer undergoing nephrectomy at the Second Hospital of Tianjin Medical University (approval number: ky2022k060). Tissue blocks free of renal cancer cells were obtained with the assistance of a pathologist. The normal kidney tissue group (Ctrl) and the nephrolithiasis group were further classified based on the presence or absence of kidney stones in the kidneys. Detailed patient information is provided in Table S1 (Supporting Information).

### Mouse Models

*Rxrα^fl^/^fl^* mice were generated using CRISPR/Cas9 (GemPharmatech Co. Ltd. with the strain number T013503) (Figure S1, Supporting Information). These mice were then crossbred with PEPCK‐Cre transgenic mice to obtain renal tubule‐specific *Rxrα* knockout mice (*Rxrα^−/−^*). Eight to ten weeks male *Rxrα^−/−^* mice and age matched wild type (WT) mice were used. All animal experimental protocols and procedures were approved by the Ethical Review Committee for Animal Experimentation of Tianjin Medical University, Tianjin, China (number SYXK: 2020‐0010). Mice were accommodated under controlled environmental conditions of a 12‐h light‐dark cycle at temperatures ranging from 21 to 25 °C, adjustable humidity levels between 30% and 70%, and unrestricted access to food and water unless otherwise specified. The mice were randomly allocated to the respective experimental groups. The position of the animal cages on the rack was random, and the mice were measured or treated in a random order. During all the stages of the animal experiment, three different investigators were involved as follows: a first investigator (Y.Y.) administered the treatment, due to overt significant weight loss the experimenter could not be blinded to whether the animal was injected with glyoxylic acid (Gly) or with saline. This investigator was the only person aware of the treatment group allocation. A second investigator (Y.S.) was responsible for the outcome assessment. Finally, a third investigator (X.D.) (unaware of treatment) performed the data analysis.

A Gly‐induced intrarenal CaOx crystal deposition mouse model was established by i.p. of Gly (G10601, Sigma–Aldrich) using a 32G needle to minimize mouse discomfort, with a dose of either 70 or 80 mg kg^−1^ per day, and mice underwent euthanasia seven days later. For JQ1 treatment, 8‐week‐old mice were injected intraperitoneally with 25 mg kg^−1^ JQ1 (S2098, Selleck) or a 10% cyclodextrin/90% saline solution daily, starting on the day of Gly injection and continuing for seven consecutive days, the sample size was calculated based on initial data assuming α = 0.05, and 1‐β = 0.9. The power calculation indicated a minimum of N = 3 in the control group and N = 8 in the experimental groups. For bexarotene treatment, 8‐week‐old mice were gavaged daily with 100 mg kg^−1^ bexarotene (S2098, Selleck) or a 10% DMSO/90% corn oil solution, the sample size was determined based on initial data, with assumptions of α = 0.05 and 1‐β = 0.9. The power calculation specified a minimum requirement of N = 3 for the control group and N = 9 for the experimental groups. Outliers were detected by identifying those with a z‐score exceeding three utilizing the outlier test. For the purposes of this manuscript, no mice were excluded.

### Isolation of Tubular Epithelial Cells (TECs)

Isolation of TECs was performed as previously described with some modifications.^[^ ^65^ ^]^ Briefly, the mouse aorta was perfused with 20 mL of cold magnetic bead solution with 40 µL Dynabeads M450 (14013, Invitrogen). The renal cortex was collected and minced into 1–3 mm tissue pieces, then rotated at 37 °C for 10 min in HBSS containing 1 mg mL^−1^ collagenase I (LS004196, Worthington), 0.75 mg mL^−1^ protease inhibitor (T6522, Sigma) and 40 U mL^−1^ DNase I (D4513, Sigma). This digestion step was performed twice, the suspension was passed through a 100 µm filter membrane to remove large tissue chunks, and then collect the filtrate and passed through a 45 µm filter membrane to eliminate the filtrate containing single‐cell components. The digested tissue on the cell filter was collected with cold PBS and washed three times in cold PBS. The filtrate was then placed on a magnetic separation rack (Promega) to remove the glomeruli. The purity of the isolated primary tubules was ≈90% (Figure S2, Supporting Information). The suspended long sections of proximal tubules were immediately used for subsequent drug screening, ChIP‐seq, and RNA‐seq analysis.

### Study Approval

This study was approved by the Ethical Committee of Tianjin Medical University (number SYXK: 2020‐0010). Human kidney tissues for this study were sourced from patients with kidney cancer undergoing nephrectomy at the Second Hospital of Tianjin Medical University (approval number: ky2022k060).

### Statistical Analysis

All data were presented as mean ± SEM. Statistical analysis was performed using SPSS 22.0 software. Student's *t*‐test was used for comparisons between two groups, and multiple groups were compared using a one‐way analysis of variance followed by the post hoc Bonferroni test. Linear correlations were analyzed using Pearson's correlation coefficient. *p* < 0.05 values were considered statistically significant.

## Conflict of Interest

The authors declare no conflict of interest.

## Author Contributions

Y.Y., X.D., and Y.S. contributed equally to this work. Y.Y. and Y.S. conducted the animal and biochemistry studies and contributed to writing the manuscript. X.D. and M.W. carried out animal studies and bioinformatic analysis, also participating in manuscript writing. J.W. and W.T. were responsible for the drug screening studies. X.C. offered expertise in bioinformatic analysis and animal studies. H.X and L.X prepared the human samples. J.N. provided insights into tissue specimen analysis and edited the manuscript. Y.C., C.L., and L.Z. conceived and supervised the project, analyzed data, and wrote the manuscript.

## Supporting information



Supporting Information

## Data Availability

The data that support the findings of this study are available in the supplementary material of this article.
