# Supplementary material for: Enhancer Profiling Reveals a Protective Role of RXRα Against Calcium Oxalate‐Induced Crystal Deposition and Kidney Injury
Source: Adv Sci (Weinh). 2025 Mar 17;12(21):2411735. doi: 10.1002/advs.202411735 (PMC12140378; doi:10.1002/advs.202411735)
Supplement: Supplementary file 1 — Supporting Information [file ADVS-12-2411735-s001.docx]

# Supplementary Table of Contents

Supplementary Table of Contents 1

Supplementary Materials and Methods 2

Supplementary Table S1: Patient information 8

Supplementary Table S2: Primers used for RT-qPCR and ChIP-qPCR analysis 9

Figure S1 11

Figure S2 12

Figure S3 13

Supplementary References 14

# Supplementary Materials and Methods

**Cell Culture**

Tubular epithelial cells (TECs) were procured from the renal tissues of C57BL/6 mice using the method previously outlined^1^. Briefly, the kidneys were perfused with magnetic beads, followed by fragmentation and digestion of the renal cortex using 1 mg/mL Type I collagenase at 37°C for a duration of 20 minutes. Glomeruli were subsequently segregated from the TECs using a magnet stand, enabling subsequent experimentation. For the drug screening process, TECs were distributed onto 96-well plates at a density of 4 000 cells per well on Day 0 and cultured until the cell population reached an estimated 70%. Subsequently, FITC-labelled crystals were incorporated at a final concentration of 5 mM, succeeded by the introduction of the drug at a concluding concentration of 10 μM, with a duplicate provision for each drug and five blank controls per plate. Following a 48-hour period, the plates underwent a triple wash with PBS to eliminate crystals not adhered to cells. The fluorescence intensity was then quantified using a high-content imaging system; specifically, the ratio between the drug well fluorescence and blank well fluorescence served to evaluate the drug's impact on crystal adhesion.

**Measurement of renal function**

Mice were terminated via euthanasia and blood was collected from cardiac puncture, followed by coagulation and centrifugation for obtaining the serum. Serum creatinine concentration was analyzed employing Jaffe’s methodology using a commercial reagent kit (BioAssay Systems, DICT-500) in accordance with the manufacturer's guidelines. Likewise, blood urea nitrogen (BUN) concentration was assessed using a separate commercial reagent kit (BioAssay Systems, DIUR-500), adhering to the provided manufacturer's instructions.

**Renal histology**

Renal specimens were subjected to an overnight fixing process in formalin before being embedded in paraffin in preparation for histological examination. Sections, with a thickness of 5 µm, underwent staining with periodic acid-Schiff, Masson’s Trichrome Stain (MTS), and further immunohistochemical staining. Tubular damage was characterized by the lack of a brush border, the presence of necrosis, intratubular casts, tubular dilation, and atrophy. The cortical tubular injury percentage was objectively determined, and the level of tubular injury was subsequently scored. The injury score was calculated as follows: (number of injured tubules/total number of tubules) × 100%. Renal tissues stained with MTS were then evaluated for the extent of interstitial fibrosis utilizing Image-Pro Plus 6.0 software.

**Detection of kidney CaOx crystals**

Kidney sections were stained with hematoxylin and eosin (H&E) following standard procedures, and Von Kossa staining method was used to detect crystal deposition in kidney tissues. Kidney crystals were quantified using ImageJ software, further statistical analysis was carried out using the ratio of the area of calcium oxalate crystals within the field of view to the entire field of view area as a score for kidney calcification.

**Immunohistochemistry**

Mouse kidneys were subjected to fixation in 10% formalin overnight, followed by paraffin embedding. Tissue sections were blocked with 3% H_2_O_2_ for 15 min and then blocked by 5% BSA for 1 hour at room temperature. The sections were then incubated overnight at 4℃ with RXRα (HUABIO, JG99-38) primary antibody. Subsequent to the primary antibody incubation, an anti-mouse/rabbit HRP secondary antibody was introduced to the sections. Imaging using a microscope (BX51, Olympus) was conducted as a final step, with all images taken using the same exposure time and gain settings.

**Western blot**

Cells were subjected to lysis in RIPA buffer (150 mM NaCl, 1.0% NP-40, 50 mM Tris-HCl pH 8.0, 1% sodium dodecyl sulfate, 0.5% sodium deoxycholate), supplemented with both protease inhibitor cocktail (Roche) and phosphatase inhibitor cocktail (Roche). Following this, SDS loading buffer was incorporated and subjected to boiling for a duration of 10 minutes. The antibodies utilized in this procedure included OPN (Santa Cruz Biotechnology, sc-73631), CD44 (Santa Cruz Biotechnology, sc-7297), RXRα (HUABIO, ET7108-99), BMP2 (Proteintech, 66383-1-lg), PPARA (Proteintech, 66826-1-lg), SLC26A1 (Proteintech, 26611-1-AP), FGG (Proteintech, 15841-1-AP) and GAPDH (Utibody, UM4002).

**RNA isolation and RT-qPCR**

Total RNA was extracted from isolated TECs, utilizing TRIzol (Invitrogen, 15596018). A total of two micrograms of RNA was then reverse-transcribed utilizing the cDNA Synthesis Kit (Roche, 4897030001) in alignment with the manufacturer’s protocol. We employed a 96-well reaction plate for SYBR-based real-time quantitative polymerase chain reaction (RT-qPCR) to discern the mRNA expression. Supplementary Table S2 provides an overview of the primer sequences.

**Low-input ChIP-seq**

The procedure for low-input ChIP-seq was conducted as previously described, with a few modifications^1^. In brief, primary TECs were distributed into tubes, each containing 200 000 cells, and were then stored on ice or at 4°C. Lysis was performed on cells in 19 μL lysis buffer, followed by incubation with 19 μL MNase buffer comprising 0.02 U MNase. This procedure was halted by adding 5 μL stop buffer. Each sample was later mixed with 45 μL 2× RIPA buffer, and permitted a stand time of 10 min before centrifugation at 14 500 rpm for 15 min. The supernatant was then relocated to a new tube and blended with 40 μL RIPA buffer. For IP samples, we added 1 μg of antibody, such as H3K4me3 (Millipore, 05-745R), H3K4me1 (Abcam, ab8895), H3K27ac (Abcam, ab4729), or RXRα (HUABIO, ET7108-99) for overnight incubation at 4 ℃. Then, Dynabeads Protein G were added and incubated for another 4 hours. Following centrifugation, the collected beads were subjected to five washes with RIPA buffer, followed by a rinse with LiCl buffer. Finally, the beads were re-suspended in 30 μL of elution buffer with the addition of 1 μL proteinase K, and were then shaken at 55°C for 90 min. The resulting supernatant, after removal, underwent heat treatment at 72°C for 40 min to inactivate proteinase K. For Input samples, each was treated with 1 μL proteinase K, followed by a 55°C shaking phase for 90 min, after which DNA was refined with DNA Extraction Reagent. Library construction was effected with a VAHTS Universal DNA Library Prep Kit for Illumina V3, and sequencing was carried out on the Illumina platform.

**ChIP-seq analysis**

Paired-end reads from ChIP-seq were aligned by the Bowtie2 algorithm against the mouse reference genome mm10. Picard (http://broadinstitute.github.io/picard/) “mark duplicates” command was applied to remove PCR duplicates and reads aligned to mtDNA were removed. MACS3 was used for each sample using fragment sizes obtained from the alignments and a *P*-value cutoff of 0.05. Then merged two biological replicates were used for further analysis. Statistically significant differences in normalized read counts across peaks between sets of ChIP-seq experiments were determined using HOMER's getDifferentialPeaks.pl, which utilizes statistical modeling functions of DESeq2. ChromHMM algorithm was applied to define the chromatin states. AnnotatePeaks.pl algorithm was used to analyze distribution of peaks around the TSS. We applied findMotifsGenome.pl algorithm for enrichment of motif and bamCoverage algorithm were used to generate bigwig file.

**RNA-seq and analysis**

Total RNAs were extracted from primary TEC using TRIzol (Invitrogen, 15596018) according to the manufacturer’s instructions. For RNA samples from Ctrl or Gly mice, libraries were constructed and sequenced on the on the Illumina NovaSeq platform. For RNA samples from WT mice treated with JQ1 or Bexarotene and and *Rxrα^-/-^* mice, libraries were constructed and sequenced on the Illumina NovaSeq platform. Sequence reads were aligned to the mm10 reference genome sequence using Hisat2. The aligned reads were sorted using samtools and read counts per gene were obtained from mapped reads using FeatureCount. Differentially expressed genes were identified by getDiffExpression.pl algorithm.

**ChIP-qPCR**

IP samples were procured as delineated in the ChIP-seq method, with the exception that the antibodies involved were SMRT (Santa Cruz Biotechnology, sc-32298) and HDAC3 (Santa Cruz Biotechnology, sc-376957). Each ChIP-qPCR reaction utilized 1 μL of purified DNA for IP samples. Concerning input samples, the purified DNA was diluted 10 times, applying the resulting 1 μL of diluted DNA per ChIP-qPCR reaction. RT-qPCR was carried out to ascertain DNA abundance, with DNA enrichment exemplified as input percentage. PCR primers were devised in accordance with ChIP-seq peaks of pertinent histones on genomic loci, and Supplementary Table S2 provides primers for ChIP-qPCR.

# Supplementary Table S1: Patient information

|  | No | Sex | Age | Creatinine (μmol/L) | BUN (mmol/L) | Stone size (cm) | Rxrα expression （H-score） |
| --- | --- | --- | --- | --- | --- | --- | --- |
| Nephrolith-iasis | 1 | F | 57 | 51.8 | 3.9 | 1.5 | 0.789 |
|  | 2 | M | 57 | 58.5 | 5.9 | 1.5 | 0.815 |
|  | 3 | F | 66 | 54.6 | 4.8 | 1.5 | 0.893 |
|  | 4 | F | 50 | 65.1 | 3.9 | 1.5 | 0.955 |
|  | 5 | M | 64 | 129 | 7 | 2.2 | 1.113 |
|  | 6 | M | 80 | 83.4 | 7.2 | 1 | 1.143 |
|  | 7 | M | 81 | 12.5 | 111.9 | 1.5 | 1.168 |
|  | 8 | M | 45 | 89.2 | 6.1 | 2 | 1.23 |
|  | 9 | M | 61 | 65.8 | 4.1 | 0.8 | 1.548 |
|  | 10 | M | 80 | 55.3 | 3.7 | 0.3 | 1.587 |
|  | 11 | M | 72 | 125.9 | 8.8 | 0.5 | 1.609 |
|  | 12 | M | 57 | 85.4 | 9.5 | 0.5 | 1.756 |
|  | 13 | M | 67 | 129.7 | 5.2 | 1 | 1.789 |
|  | 14 | F | 59 | 49.9 | 5.4 | 1 | 1.824 |
|  | 15 | M | 72 | 90.6 | 6.8 | 0.3 | 1.91 |
|  | 16 | F | 67 | 92.5 | 3.7 | 2 | 0.16 |
|  | 17 | F | 55 | 73.2 | 5.9 | 2 | 0.579 |
|  | 18 | F | 60 | 146.8 | 10.4 | 1.5 | 0.689 |
|  | 19 | F | 66 | 79.4 | 5.6 | 1 | 0.764 |
| Ctrl | 20 | M | 61 | 77.8 | 6 | NA | 1.5 |
|  | 21 | M | 64 | 84.4 | 4.7 | NA | 1.647 |
|  | 22 | F | 58 | 66.3 | 4.9 | NA | 1.714 |
|  | 23 | M | 58 | 128.8 | 7.4 | NA | 1.789 |
|  | 24 | F | 60 | 74.2 | 6.3 | NA | 1.818 |
|  | 25 | M | 80 | 100.2 | 10 | NA | 1.879 |
|  | 26 | M | 45 | 86.9 | 3.6 | NA | 1.919 |
|  | 27 | F | 67 | 100.2 | 6 | NA | 1.927 |
|  | 28 | F | 60 | 82.8 | 5.3 | NA | 1.941 |
|  | 29 | M | 57 | 422.6 | 21 | NA | 1.959 |
|  | 30 | F | 65 | 46.7 | 3.6 | NA | 1.982 |
|  | 31 | F | 50 | 63.1 | 5.3 | NA | 2.002 |
|  | 32 | F | 56 | 56.7 | 4.7 | NA | 2.082 |
|  | 33 | M | 72 | 61.9 | 4.8 | NA | 2.135 |
|  | 34 | F | 57 | 88.1 | 6.1 | NA | 2.149 |
|  | 35 | F | 58 | 52 | 4.3 | NA | 2.15 |
|  | 36 | M | 59 | 77.7 | 7.2 | NA | 2.375 |

# Supplementary Table S2: Primers used for RT-qPCR and ChIP-qPCR analysis

| Gene | DNA sequence (5’ > 3’) |
| --- | --- |
| Primers used for RT-qPCR | |
| *Pparα* F | TGCCTTCCCTGTGAACTGAC |
| *Pparα* R | TTCGCCGAAAGAAGCCCTTA |
| *Slc26a1* F | CTAGTGCCTGGTGAGACACA |
| *Slc26a1* R | AGGCTCTTTGGCTTATCTGTT |
| *Spp1* F | CTGGCTGAATTCTGAGGGACT |
| *Spp1* R | ACAGGGATGACATCGAGGGA |
| *Cd44* F | AGAAGGGACAACTGCTTCGG |
| *Cd44* R | GTGGTCACTCCACTGTCCTG |
| *Bmp2* F | TTCCATCACGAAGAAGCCGT |
| *Bmp2* R | GGCCGTTTTCCCACTCATCT |
| *Fgg* F | GCACCACAGAGTTTTGGCTG |
| *Fgg* R | ATAGTCCGCAGTGCTGGTTC |
| *Sp7* F | AAGAGTGAGCTGGCCTGAGA |
| *Sp7* R | CCATAGTGAGCTTCTTCCTGGG |
| *Stat3* F | GACATTCCCAAGGAGGAGGC |
| *Stat3* R | TACGGGGCAGCACTACCT |
| *Gapdh* F | ACCCTTAAGAGGGATGCTGC |
| *Gapdh* R | CCCAATACGGCCAAATCCGT |
| Primers used for ChIP-qPCR | |
| *Bmp2* ChIP-qPCR F | AACAGAAGCGTTTGCTCACA |
| *Bmp2* ChIP-qPCR R | CGGGAGACTAGGGCAAATCC |
| *Sp7* ChIP-qPCR F | GTCGGGTCTTTCTCTGCCTC |
| *Sp7* ChIP-qPCR R | TTCCTCAGGTTGTTGCAGGG |
| *Stat3* ChIP-qPCR F | TGACTCATGGGAGCTAGGTTGT |
| *Stat3* ChIP-qPCR R | TCACAGTTACCCTTCACTTGATGT |

# Figure S1

#
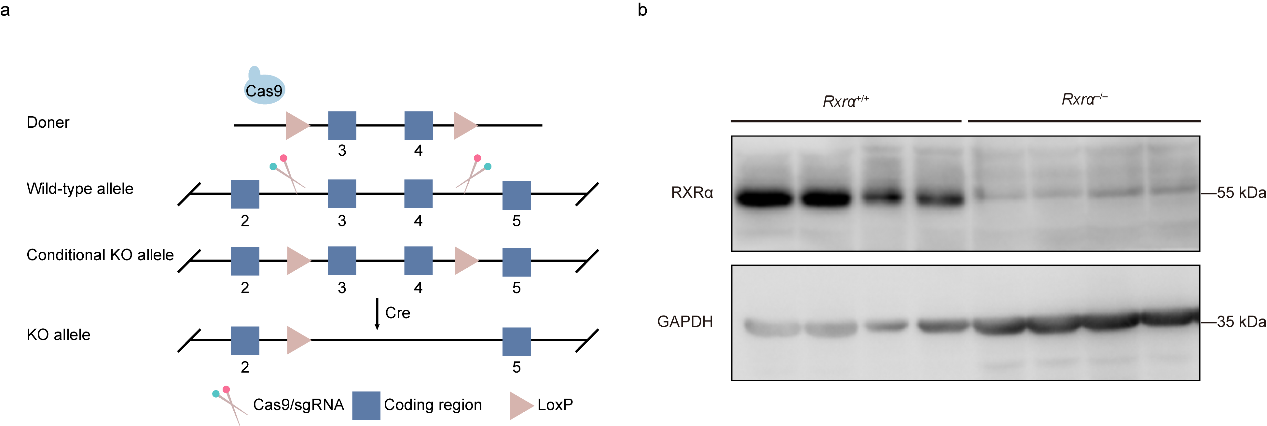


**Figure S1. Generation and characterization of *Rxrα*-knockout mouse.**

**(a)** Schematic diagram of tubule-specific *Rxrα* knockout mice generation using the CRISPR-Cas9 system. **(b)** Western blot analysis of RXRa in TECs from WT and *Rxrα* knockout mice.

# Figure S2


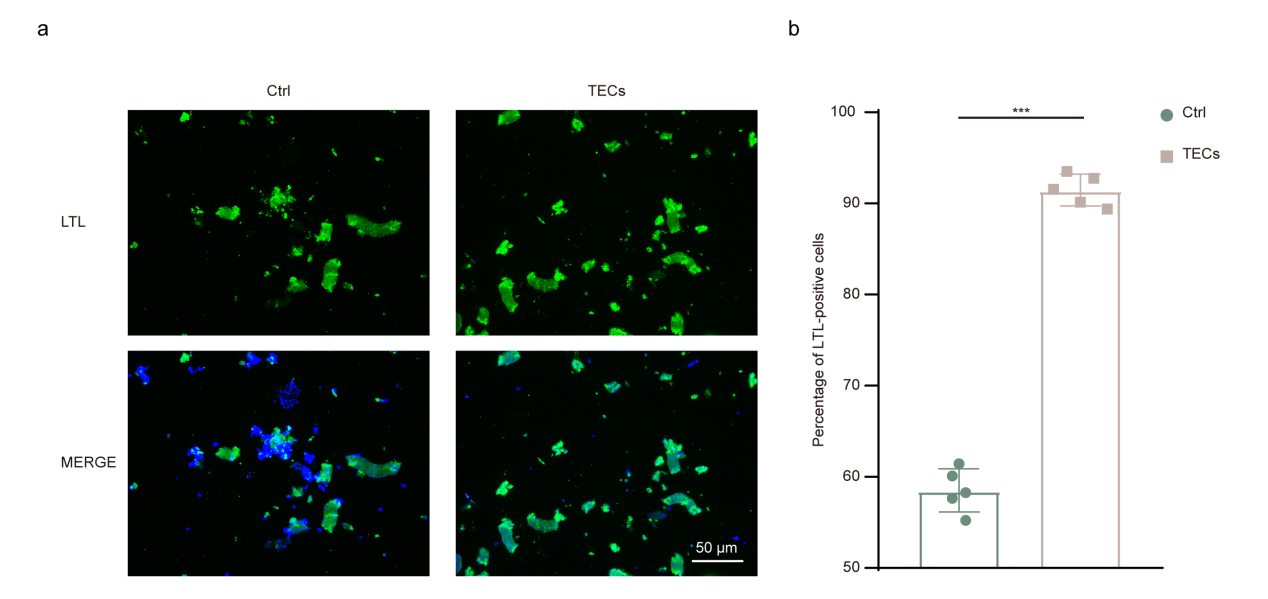


**Figure S2. Pure primary tubular cells were obtained after sorting.**

**(a)** Analysis of LTL staining in whole renal cells (Ctrl) and primary TECs. **(b)** quantification of LTL positive cells. Scale bars: 50 μm (A). Data presented as mean ± SEM. ****P* < 0.001.

# Figure S3


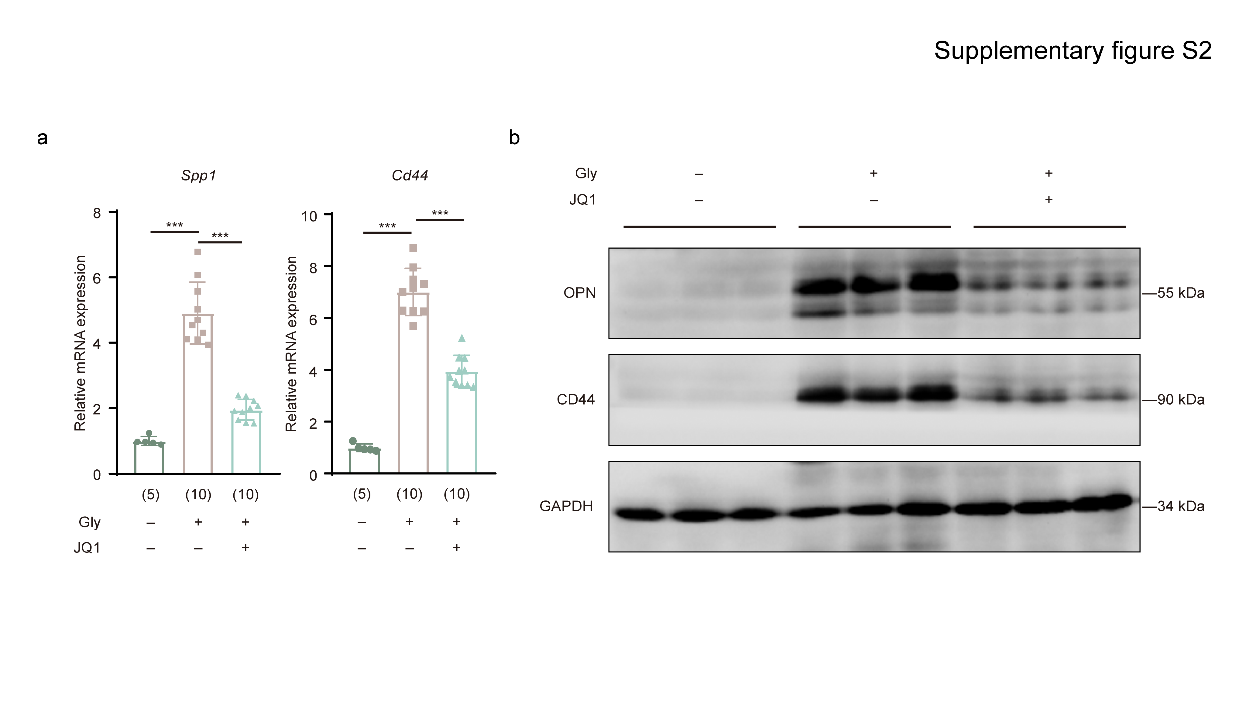


**Figure S3.** **JQ1 reduced the expression of nephrolithiasis-promoting genes.**

**(a)** RT-qPCR analysis of *Spp1* (left) and *Cd44* (right) expression in the indicated groups. **(b)** Western blot analysis of OPN and CD44 in TECs from intrarenal CaOx crystal deposition mice treated with JQ1. Data presented as mean ± SEM. ****P* < 0.001.

# Supplementary References

1. Ji R, Chen J, Xie Y, et al. Multi-omics profiling of cholangiocytes reveals sex-specific chromatin state dynamics during hepatic cystogenesis in polycystic liver disease. *J Hepatol*. Apr 2023;78(4):754-769.
